# Efficient PbS/CdS co-sensitized solar cells based on TiO_2_ nanorod arrays

**DOI:** 10.1186/1556-276X-8-67

**Published:** 2013-02-11

**Authors:** Yitan Li, Lin Wei, Xiya Chen, Ruizi Zhang, Xing Sui, Yanxue Chen, Jun Jiao, Liangmo Mei

**Affiliations:** 1 School of Physics, State Key Laboratory of Crystal Materials, Shandong University, Jinan, 250100, People’s Republic of China; 2 School of Information Science and Engineering, Shandong University, Jinan, 250100, People’s Republic of China; 3 Department of Mechanical and Materials Engineering, Portland State University, P.O. Box 751, Portland, OR, 97207-0751, USA; 4 Department of Physics, Portland State University, P.O. Box 751, Portland, OR, 97207-0751, USA

**Keywords:** TiO_2_, PbS, CdS, Nanorod, Solar cells

## Abstract

Narrow bandgap PbS nanoparticles, which may expand the light absorption range to the near-infrared region, were deposited on TiO_2_ nanorod arrays by successive ionic layer adsorption and reaction method to make a photoanode for quantum dot-sensitized solar cells (QDSCs). The thicknesses of PbS nanoparticles were optimized to enhance the photovoltaic performance of PbS QDSCs. A uniform CdS layer was directly coated on previously grown PbS-TiO_2_ photoanode to protect the PbS from the chemical attack of polysulfide electrolytes. A remarkable short-circuit photocurrent density (approximately 10.4 mA/cm^2^) for PbS/CdS co-sensitized solar cell was recorded while the photocurrent density of only PbS-sensitized solar cells was lower than 3 mA/cm^2^. The power conversion efficiency of the PbS/CdS co-sensitized solar cell reached 1.3%, which was beyond the arithmetic addition of the efficiencies of single constituents (PbS and CdS). These results indicate that the synergistic combination of PbS with CdS may provide a stable and effective sensitizer for practical solar cell applications.

## Background

Quantum dot-sensitized solar cells can be regarded as a derivative of dye-sensitized solar cells, which have attracted worldwide scientific and technological interest since the breakthrough work pioneered by O’Regan and Grätzel [1-5]. Although the light-to-electric conversion efficiency of 12% [6] reported recently was very impressive, the use of expensive dye to sensitize the solar cell is still not feasible for practical applications. Therefore, it is critical to tailor the materials to be not only cost-effective but also long lasting. Inorganic semiconductors have several advantages over conventional dyes: (1) The bandgap of semiconductor nanoparticles can be tuned by size to match the solar spectrum. (2) Their large intrinsic dipole moments can lead to rapid charge separation and large extinction coefficient, which is known to reduce the dark current and increase the overall efficiency. (3) In addition, semiconductor sensitizers provide new chances to utilize hot electrons to generate multiple charge carriers with a single photon. Hence, nanosized narrow bandgap semiconductors are ideal candidates for the optimization of a solar cell to achieve improved performance.

Recently, various nanosized semiconductors including CdS [7], CdSe [8], CuInS_2_[9], Sb_2_S_3_[10,11], PbS [12], as well as III-VI quantum ring [13,14] have been studied for solar cell applications. Among these nanomaterials, lead sulfide (PbS) has shown much promise as an impressive sensitizer due to its reasonable bandgap of about 0.8 eV in the bulk material, which can allow extension of the absorption band toward the near infrared (NIR) part of the solar spectrum. Recently, Sambur et al. experimentally demonstrated the collection of photocurrents with quantum yields greater than one electron per photon in the PbS QD-sensitized planar TiO_2_ single crystal utilizing polysulfide electrolyte, which is undoubtedly encouraging to the future photovoltaic development [15]. Furthermore, PbS has a large exciton Bohr radius of about 20 nm, which can lead to extensive quantum size effects. It has been reported that its absorption range can be tuned by adjusting the particle size of the quantum dots [16,17]. Until now, as one of the most impressive alternative semiconductors, PbS-sensitized solar cells have been studied by many groups [18-22]. In most of the reported works, PbS quantum dots were grown on TiO_2_ nanotubes [20], ZnO nanorod arrays [21], and TiO_2_ photoanode with hierarchical pore distribution [22]. Little work has been carried out on large-area single-crystalline TiO_2_ nanorod array photoanode. Compared to the polycrystal TiO_2_ nanostructures such as nanotubes [23] and nanoparticles [24], single-crystalline TiO_2_ nanorods grown directly on transparent conductive oxide electrodes provide a perfect solution by avoiding the particle-to-particle hopping that occurs in polycrystalline films, thereby increasing the photocurrent efficiency. In addition to the potential of improving electron transport, they enhance light harvesting by scattering the incident light.

In this paper, narrow bandgap PbS nanoparticles and single-crystalline rutile TiO_2_ nanorod arrays were combined to produce a practical semiconductor-sensitized solar cell. Several sensitizing configurations have been studied, which include the deposition of ‘only PbS’ or ‘only CdS’ and the hybrid system PbS/CdS. Optimized PbS SILAR cycle was obtained, and the uniformly coated CdS layer can effectively minimize the chemical attack of polysulfide electrolytes on PbS layer. Therefore, the performance of sensitized solar cells was stabilized and long lasting. The power conversion efficiency of PbS/CdS co-sensitized solar cell showed an increase of approximately 500% compared with that sensitized by only PbS nanoparticles.

## Methods

### Growth of TiO_2_ nanorod arrays by hydrothermal process

The TiO_2_ nanorod arrays were grown directly on fluorine-doped tin oxide (FTO)-coated glass using the following hydrothermal methods: 50 mL of deionized water was mixed with 40 mL of concentrated hydrochloric acid. After stirring at ambient temperature for 5 min, 400 μL of titanium tetrachloride was added to the mixture. The mixture was injected into a stainless steel autoclave with a Teflon container cartridge. The FTO substrates were ultrasonically cleaned for 10 min in a mixed solution of deionized water, acetone, and 2-propanol with volume ratios of 1:1:1 and were placed at an angle against the Teflon container wall with the conducting side facing down. The hydrothermal synthesis was conducted at 180°C for 2 h.After synthesis, the autoclave was cooled to room temperature under flowing water, and the FTO substrates were taken out, rinsed thoroughly with deionized water, and dried in the open air.

### Deposition of PbS and CdS layers with successive ionic layer adsorption and reaction method

In a typical SILAR cycle for the deposition of PbS nanparticles, the FTO conductive glass, pre-grown with TiO_2_ nanorod arrays, was dipped into the 0.02 M Pb(NO_3_)_2_ methanol solution for 2 min then dipped into 0.02 M Na_2_S solution (obtained by dissolving Na_2_S in methanol/water with volume ratios of 1:1) for another 5 min. This entire SILAR process was repeated from 1 to 10 cycles to achieve the desired thickness of PbS nanoparticle layer. Similarly, for the CdS nanoparticle layer, Cd^2+^ ions were deposited from a 0.05 M Cd(NO_3_)_2_ ethanol solution, and the sulfide sources were 0.05 M Na_2_S in methanol/water (50/50 *v*/*v*). For the hybrid PbS/CdS co-sensitized samples, the CdS deposition was carried out immediately after PbS deposition. The samples are labeled as PbS(*X*)/CdS(*Y*)-TiO_2_, where *X* and *Y* refer to the number of PbS and CdS SILAR cycles, respectively.

### Characterization

The crystal structure of the CdS-TiO_2_ and PbS-TiO_2_ samples were examined by X-ray diffraction (XRD; XD-3, PG Instruments Ltd., Beijing, China) with Cu Kα radiation (*λ* = 0.154 nm) at a scan rate of 2°/min. X-ray tube voltage and current were set at 40 kV and 30 mA, respectively. The surface morphology and the cross section of the CdS-TiO_2_, PbS-TiO_2_, and PbS/CdS-TiO_2_ nanostructures were examined by a field-emission scanning electron microscopy (FESEM; FEI Sirion, FEI Company, Hillsboro, OR, USA).

### Solar cell assembly and performance measurement

The solar cells were assembled using the CdS-TiO_2_, PbS-TiO_2_, and PbS/CdS-TiO_2_ nanostructures as the photoanodes, respectively. Pt counter electrodes were prepared by depositing 20-nm Pt film on FTO glass using a magnetron sputtering. A 60-μm-thick sealing material (SX-1170-60, Solaronix SA, Aubonne, Switzerland) was pasted onto the Pt counter electrodes. The Pt counter electrode and a nanostructure photoanode were sandwiched and sealed with the conductive sides facing inward. A polysulfide electrolyte was injected into the space between two electrodes. The polysulfide electrolyte was composed of 0.1 M sulfur, 1 M Na_2_S, and 0.1 M NaOH, which were dissolved in methanol/water (7:3 *v*/*v*) and stirred at 60°C for 1 h.

A solar simulator (model 94022A, Newport, OH, USA) with an AM1.5 filter was used to illuminate the working solar cell at light intensity of 1 sun (100 mW/cm^2^). A sourcemeter (2400, Keithley Instruments Inc., Cleveland, OH, USA) was used for electrical characterization during the measurements. The measurements were carried out with respect to a calibrated OSI standard silicon solar photodiode.

## Results and discussion

### Morphology and crystal structure of the nanostructured photoanodes

**Figure 1:**
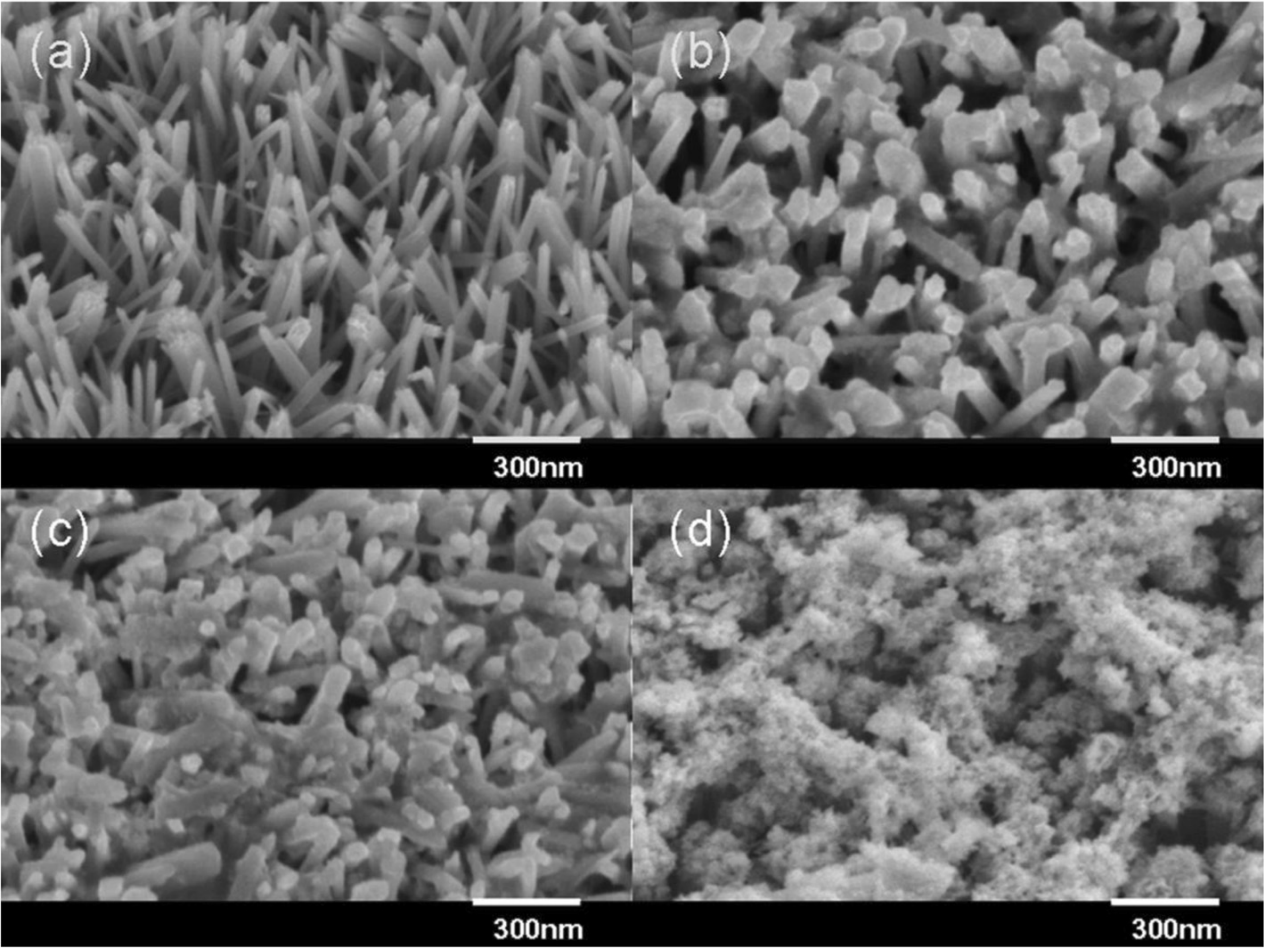
**Typical FESEM images of the bare TiO**_**2 **_**nanorod array and PbS-TiO**_**2 **_**nanostructures.** (**a**) FESEM image (40° tilted) of the bare TiO_2_ nanorod array grown on FTO glass by hydrothermal method. (**b**) FESEM images of PbS-TiO_2_ nanostructures after 1, (**c**) 3, and (**d**) 5 SILAR cycles.

Figure 1a shows the typical FESEM images of TiO_2_ nanorod arrays on an FTO-coated glass substrate, confirming that the FTO-coated glass substrate was uniformly covered with ordered TiO_2_ nanorods. The density of nanorods was approximately 20 nanorods/μm^2^ with suitable space for deposition of PbS and CdS nanoparticles. Figure 1b,c,d shows TiO_2_ nanorods coated by PbS nanoparticles after 1, 3, 5 SILAR cycles, respectively. With the increase of SILAR cycles, the thickness of the PbS nanoparticles increased correspondingly. For the sample coated with 5 SILAR cycles, the space between the TiO_2_ nanorods was filled with PbS nanoparticles, and a porous PbS nanoparticle layer was formed on the surface of the TiO_2_ nanorods. As discussed later, this porous PbS layer can cause a dramatic decrease in photocurrent and efficiency for the solar cells.

**Figure 2:**
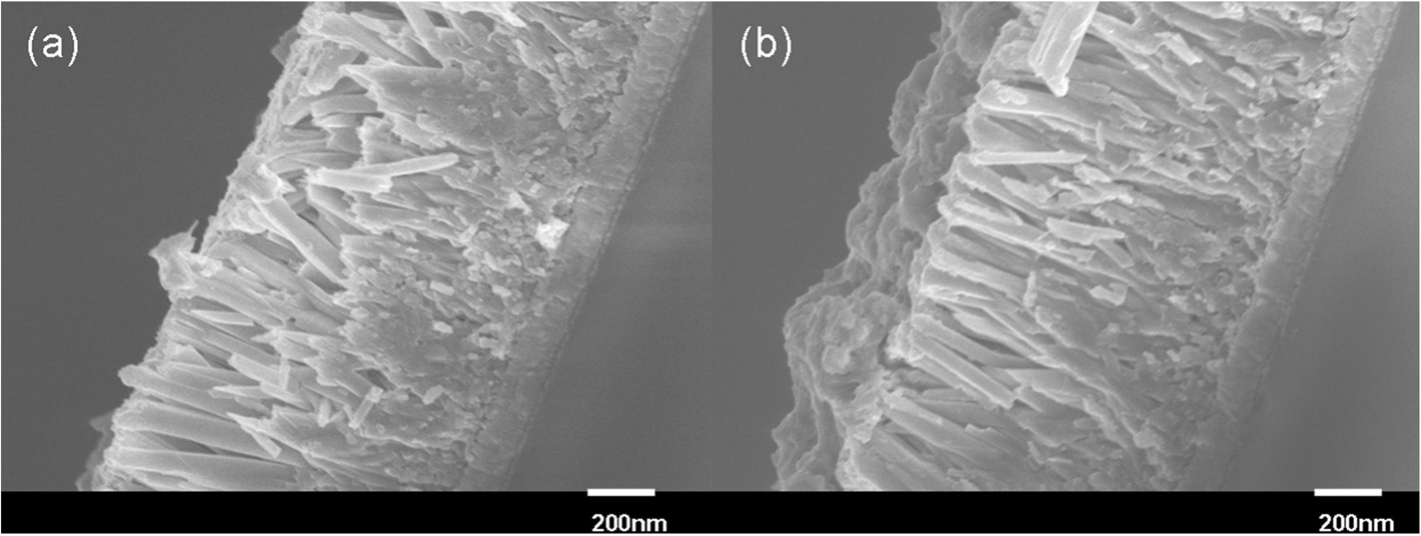
**Cross-sectional SEM images of PbS-TiO**_**2**_**nanostructures without (a) and with (b) CdS capping layer.**

**Figure 3:**
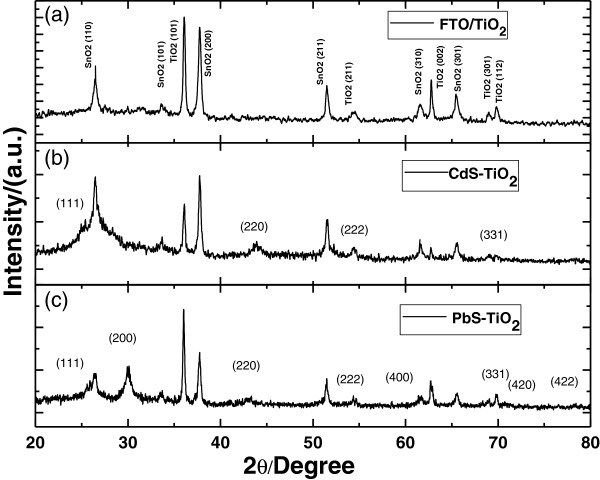
**XRD patterns of bare TiO**_**2**_**nanorod array (a), CdS-TiO**_**2**_**nanostructure (b), and PbS-TiO**_**2**_**nanostructure (c).**

Figure 2 shows the cross-sectional SEM images of PbS(3)/CdS(0)-TiO_2_ and PbS(3)/CdS(10)-TiO_2_ nanostructures. Compared with Figure 2a, a uniform protective layer of CdS was successfully deposited on the top of PbS nanoparticles. As we will discuss later, after the CdS coating, a remarkable enhancement of the cell performance and the photochemical stabilization of PbS sensitizer was observed. XRD patterns of the bare TiO_2_ nanorod array, the PbS(3)/CdS(0)-TiO_2_ nanostructure, and PbS(0)/CdS(10)-TiO_2_ nanostructure were shown in Figure 3. As shown in Figure 3a, besides the diffraction peaks from cassiterite on structured SnO_2_, all the other peaks could be indexed as the (101), (211), (002), (310), and (112) planes of tetragonal rutile structure TiO_2_ (JCPDS no.02-0494). The formation of rutile TiO_2_ nanorod arrays could be attributed to the small lattice mismatch between FTO and rutile TiO_2_[25]. Both rutile and SnO_2_ have near identical lattice parameters with *a* = 0.4594, *c* = 0.2958, and *a* = 0.4737, *c* = 0.3185 nm for TiO_2_ and SnO_2_, respectively, making the epitaxial growth of rutile TiO_2_ on FTO film possible. On the other hand, anatase and brookite have lattice parameters of *a* = 0.3784, *c* = 0.9514 and *a* = 0.5455, *c* = 0.5142 nm, respectively. The production of these phases is unfavorable due to a very high activation energy barrier which cannot be overcome at the low temperatures used in this hydrothermal reaction. As noted in Figure 3b,c, the as-synthesized CdS-TiO_2_ nanostructure exhibited weak diffraction peaks of CdS at 2*θ* = 26.5°, 43.9°, 54.6°, and 70.1°, corresponding to the (111), (220), (222), and (331) planes of cubic CdS with the lattice constant *a* = 0.583 nm (JCPDS no. 89–0440). The diffraction peaks of as-synthesized PbS-TiO_2_ nanostructure could be indexed as (111), (200), (220), (222), (400), (331), (420), and (422) planes, correspondingly, of cubic PbS with the lattice constant *a* = 0.593 nm (JCPDS no. 78–1901).

### Photovoltaic performance of PbS/CdS-TiO_2_ nanostructured solar cells

**Figure 4:**
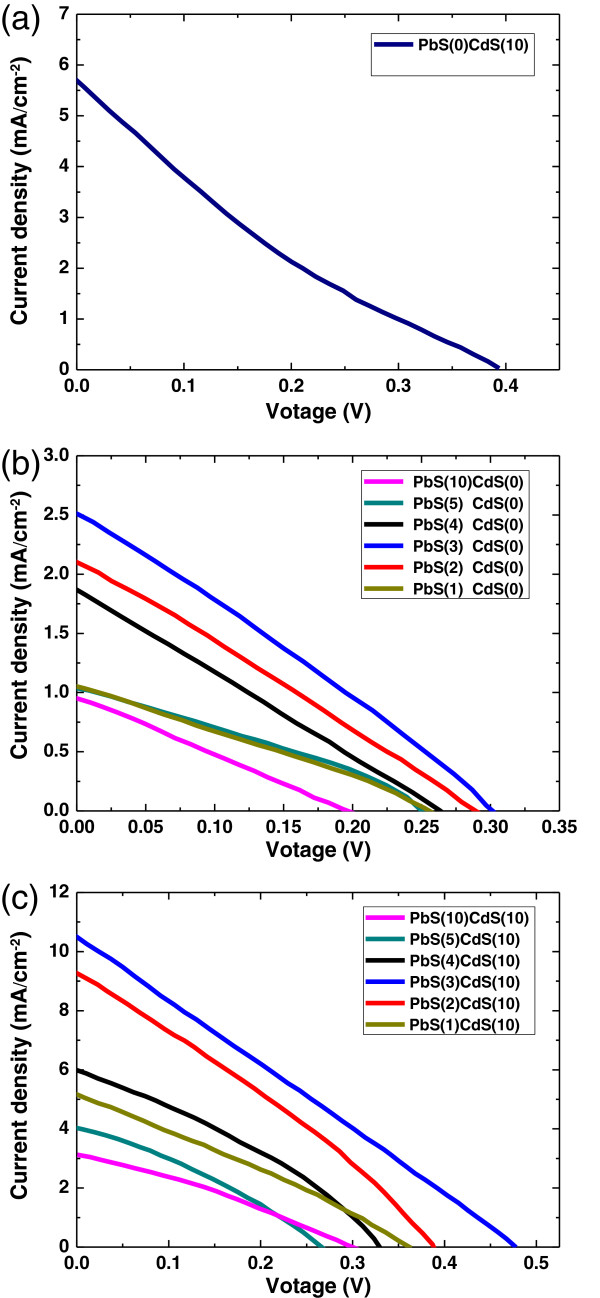
**Photovoltaic performance of PbS/CdS co-sensitized solar cells.** (**a**) Photocurrent density-voltage characteristic for only CdS-sensitized solar cell and (**b**) only PbS-sensitized solar cell. (**c**) Photocurrent density-voltage characteristic for PbS/CdS co-sensitized solar cells with different PbS SILAR cycles.

Figure 4 showed the photocurrent-voltage (*I*-*V*) performance of the sensitized solar cells assembled using PbS/CdS-TiO_2_ nanostructured photoanodes. All the photocurrent-voltage performance parameters were summarized in Table 1. Solar cell sensitized by only CdS exhibits a short-circuit photocurrent density (*J*_SC_) of 5.7 mA/cm^2^ and an open-circuit voltage (*V*_OC_) of 0.39 V. On the other hand, solar cell sensitized by only PdS present a poor photovoltaic performance with very low *J*_SC_ and *V*_OC_. Optimal PbS SILAR cycles on this photoanode were investigated. As we can see from Figure 4b, with the increase of PbS SILAR cycles, a non-monotonic change of both *J*_SC_ and *V*_OC_ is recorded. Both *J*_SC_ and *V*_OC_ of the PbS-sensitized solar cells increase with the SILAR cycles first, and a maximum *J*_SC_ of 2.5 mA/cm^2^ and *V*_OC_ of 0.3 V are obtained for the sample with 3 SILAR cycles. With further increasing PbS SILAR cycles, *J*_SC_ and *V*_OC_ decrease simultaneously, which demonstrates that a thick Pbs nanoparticles layer may hinder PbS regeneration by the electrolyte and enhance the recombination reaction. During the measurement, a continuous decrease of the current was observed, indicating the progressive degradation of PbS, which can be reasonably attributing to PbS oxidative processes. To protect the PbS nanoparticles from the chemical attack by polysulfide electrolytes, a uniform CdS layer was capped on the PbS-TiO_2_ photoanode to avoid the direct contact of PbS with the polysulfide electrolyte. As shown in Figure 4c, under the same PbS deposition cycles, the cell with CdS capping layer presents both increased *J*_SC_ and *V*_OC_, indicating that CdS QDs is indispensable to highly efficient PbS-sensitized solar cells. With the appearance of CdS layer, *J*_SC_ of the cell with 3 PbS SILAR cycles was improved from about 2.5 to 10.4 mA/cm^2^, and the *V*_oc_ was increased from 0.3 to 0.47 V. The cell efficiency reached a promising 1.3%, indicating a five times increase, which is beyond the arithmetic addition of the efficiencies of single constituents (PbS and CdS). In addition to the increase of the cell performance for the co-sensitized configurations, a significant increase of the photochemical stability of PbS takes place with the presence of the CdS coating.

**Table 1:** ***J***_**sc**_**,*V***_**oc**_**, FF, and efficiency**

	***V***_**oc**_**(V)**	***J***_**SC**_**(mA/cm**^**2**^**)**	**FF (%)**	***η*(%)**
PbS(0)CdS(10)	0.39	6.26	0.18	0.44
PbS(10)CdS(0)	0.19	0.91	0.29	0.05
PbS(5)CdS(0)	0.25	1.12	0.25	0.07
PbS(4)CdS(0)	0.26	1.83	0.27	0.13
PbS(3)CdS(0)	0.29	2.48	0.27	0.20
PbS(2)CdS(0)	0.28	2.11	0.27	0.16
PbS(1)CdS(0)	0.25	1.10	0.29	0.08
PbS(10)CdS(10)	0.30	3.12	0.29	0.28
PbS(5)CdS(10)	0.26	3.98	0.33	0.34
PbS(4)CdS(10)	0.33	5.88	0.31	0.61
PbS(3)CdS(10)	0.47	10.40	0.27	1.30
PbS(2)CdS(10)	0.39	9.09	0.30	1.05
PbS(1)CdS(10)	0.36	5.24	0.24	0.46

*V*_oc_, open-circuit voltage; *J*_sc_, short-circuit photocurrent density; FF, fill factor; *η*, energy conversion efficiency.

With further improvement of their performance, this kind of PbS/CdS co-sensitized TiO_2_ nanorod solar cells may play a promising role in the future due to the following reasons: (1) The bandgap of PbS nanoparticles is quite small and extends the absorption band towards the NIR part of the solar spectrum, which will result in a high current density. (2) TiO_2_ nanorod arrays grown directly on FTO conductive glass avoid the particle-to-particle hopping that occurs in polycrystalline mesoscopic TiO_2_ films, which can also contribute to a higher efficiency. (3) TiO_2_ nanorods form a relatively open structure, which is advantageous over the diffusion problems associated with the redox couples in porous TiO_2_ network.

In our present work, the cell efficiency was still not high enough for practical application. The drawback limiting the energy conversion efficiency of this type of solar cells was the rather poor fill factor. This low fill factor may be ascribed to the lower hole-recovery rate of the polysulfide electrolyte, leading to a higher probability for charge recombination [26]. To further improve the efficiencies of these PbS/CdS-TiO_2_ nanostructured solar cells, a new hole transport medium with suitable redox potential and low electron recombination at the semiconductor-electrolyte interface should be developed. Counter electrode was another important factor influencing the energy conversion efficiency. Recently, Sixto et al. [27] and Seol et al. [28] reported that the fill factor was clearly influenced by counter electrode materials where Au, CuS_2_, and carbon counter electrode show better performance than Pt ones. Moreover, deposition of a ZnS passivation layer on the photoanode after the PbS/CdS sensitization would greatly eliminate interfacial charge recombination and improve the photovoltaic performance of PbS/CdS-TiO_2_ nanostructured solar cells [29]. Further work to improve the photovoltaic performance of these solar cells is currently under investigation.

## Conclusion

In this study, large-area ordered rutile TiO_2_ nanorod arrays were utilized as photoanodes for PbS/CdS co-sensitized solar cells. Narrow bandgap PbS nanoparticles dramatically increase the obtained photocurrents, and the CdS capping layer stabilizes the solar cell behavior. The synergistic combination of PbS with CdS provides a stable and effective sensitizer compatible with polysulfide. Compared to only PbS-sensitized solar cells, the cell power conversion efficiency was improved from 0.2% to 1.3% with the presentation of a CdS protection layer. The PbS/CdS co-sensitized configuration has been revealed to enhance the solar cell performance beyond the arithmetic addition of the efficiencies of the single constituents. In this sense, PbS and CdS constitute a promising nanocomposite sensitizer with supracollecting properties for practical solar cell applications.

## Competing interests

The authors declare that they have no competing interests.

## Authors’ contributions

The work presented here was performed in collaboration of all authors. YL carried out the deposition of PbS and CdS layers and solar cell assembly, and drafted the manuscript. LW carried out the XRD and SEM characterizations. XC carried out the photovoltaic performance measurements. RZ and XS carried out the preparation of TiO_2_ nanorod arrays. YC supervised the work and finalized the manuscript. JJ and LM proofread the manuscript and polished the language. All authors read and approved the final manuscript.
